# Exploring adolescent girls and young women's PrEP‐user profiles: qualitative insights into differentiated PrEP delivery platform selection and engagement in Cape Town, South Africa

**DOI:** 10.1002/jia2.26254

**Published:** 2024-05-02

**Authors:** Elzette Rousseau, Kathleen J. Sikkema, Robin F. Julies, Katelyn Mazer, Gabrielle O'Malley, Renee Heffron, Jennifer F. Morton, Rachel Johnson, Connie Celum, Jared M. Baeten, Linda‐Gail Bekker

**Affiliations:** ^1^ Desmond Tutu HIV Centre University of Cape Town Cape Town South Africa; ^2^ Department of Sociomedical Sciences Mailman School of Public Health Columbia University New York City New York USA; ^3^ Department of Psychology University of the Western Cape Cape Town South Africa; ^4^ Department of Epidemiology Fielding School of Public Health University of California Los Angeles California USA; ^5^ Department of Global Health Medicine and Epidemiology University of Washington Seattle Washington USA; ^6^ Department of Medicine Heersink School of Medicine University of Alabama at Birmingham Birmingham Alabama USA

**Keywords:** adolescent girls and young women, courier, HIV self‐testing, mobile clinic, pre‐exposure prophylaxis, youth club

## Abstract

**Introduction:**

Adolescent girls and young women (AGYW), a priority population for HIV prevention in Africa, show high interest but difficulty in sustained effective use of pre‐exposure prophylaxis (PrEP). With ongoing PrEP scale‐up focused on increasing access, it is important to understand what influences AGYW's choice of PrEP delivery platforms.

**Methods:**

The POWER implementation study in Cape Town provided PrEP between 2017 and 2020 to AGYW (16−25 years) from four differentiated delivery platforms: mobile clinic, government facility, courier delivery or community‐based youth club. Healthcare providers at government and mobile clinics provided PrEP (initiation and refills) as part of comprehensive, integrated sexual and reproductive health services. Courier and youth club platforms provided light‐touch PrEP refill services incorporating rapid HIV self‐testing. We conducted in‐depth interviews with a purposive sample of AGYW who had ≥3 months of PrEP‐use and accessed ≥2 PrEP delivery platforms. The thematic analysis explored AGYW's preferences, decision‐making and habits related to PrEP access to inform market segmentation.

**Results:**

We interviewed 26 AGYW (median age 20) PrEP‐users between November 2020 and March 2021. AGYW PrEP‐users reported accessing different services with, 24 accessing mobile clinics, 17 courier delivery, 9 government health facilities and 6 youth clubs for their PrEP refills. Qualitative findings highlighted four potential behavioural profiles. The “Social PrEP‐user” preferred PrEP delivery in peer spaces, such as youth clubs or adolescent‐friendly mobile clinics, seeking affirmation and social support for continued PrEP use. The “Convenient PrEP‐user” favoured PrEP delivery at easily accessible locations, providing quick (courier) or integrated contraception‐PrEP refill visits (mobile and government clinic). The “Independent PrEP‐user” preferred PrEP delivery that offered control over delivery times that fit into their schedule, such as the courier service. The “Discreet PrEP‐user” highly valued privacy regarding their PrEP use (courier delivery) and avoided delivery options where unintentional disclosure was evident (youth club). Comfort with HIV self‐testing had minimal influence on PrEP delivery choice.

**Conclusions:**

Market segmentation of AGYW characterizes different types of PrEP‐users and has the potential to enhance tailored messaging and campaigns to reach specific segments, with the aim of improving sustained PrEP use and HIV prevention benefits.

## 1 INTRODUCTION

The past 5 years have seen the global inclusion of oral pre‐exposure prophylaxis (PrEP) into national guidelines as a key biomedical HIV prevention method, with many countries planning to deliver services that are integrated, differentiated, and sometimes digitalized for optimal PrEP use [1]. In the South African context, adolescent girls and young women (AGYW) are a group with high HIV vulnerability, high interest and uptake of PrEP, but difficulty in continued use [2]. Frequently reported barriers to effective PrEP use in AGYW include community‐level HIV‐related stigma, relational barriers, such as gender‐based violence, disclosure concerns and lack of social support, and access barriers, including inconvenient locations, times of PrEP services and judgemental interactions with healthcare providers [2, 3, 4, 5, 6, 7, 8, 9, 10, 11]. There has been a growing trend towards tailoring PrEP delivery, counselling, and support more closely to individuals’ circumstances and needs to improve uptake and use.

In scaling up PrEP delivery, differentiated services (including non‐clinic‐based services) have been called for to increase access to PrEP for those who need it most. The World Health Organization released updated guidelines in 2022 recommending simplified, demedicalized, differentiated PrEP delivery services, including the use of HIV self‐testing (HIVST), that is person‐ and community‐centred [1]. A systematic review of discreet choice experiments in key populations, including AGYW, has indicated that the most important attributes of optimal PrEP access included cost, PrEP delivery outside of HIV treatment sites, PrEP services integrated with contraception provision, HIV testing that is quick with results available immediately, and services conveniently located and at times outside of regular clinic hours [12, 13, 14].

While research has highlighted the need for biomedical PrEP products to fit into AGYW's lifestyles and relationship dynamics [15, 16, 17], optimized HIV prevention outcomes will also require matching PrEP service delivery platforms with AGYW's unique needs and diverse habits, routines and way of living. Consumer segmentation is a widely used technique within marketing to align the demand and supply of services to groups of individuals with shared priorities and needs based on their behaviour, attitudes and beliefs [18, 19, 20]. Market segmentation has previously been used in family planning counselling and for HIV prevention through voluntary medical male circumcision [21, 22, 23]. The POWER (Prevention Options for Women Evaluation Research) study provided PrEP to AGYW in Cape Town, South Africa, via differentiated PrEP delivery models, which included initiation at a mobile clinic or government health facility, with follow‐up and PrEP refills offered at four platforms, including the mobile clinic, government health facility, as well as via a youth PrEP club, and courier delivery. In relation to the POWER study, we explored AGYW PrEP‐user preferences, decision‐making, influences and habits related to PrEP access from different delivery platforms to inform market segmentation with the purpose of universal and increased access to all AGYW who may benefit from PrEP.

## 2 METHODS

### 2.1 Research setting and study participants

**Figure 1:**
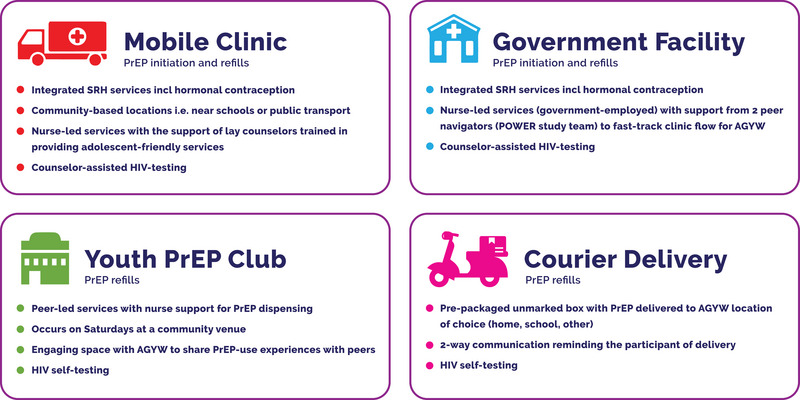
POWER study differentiated PrEP delivery platform for AGYW. AGYW, adolescent girls and young women; PrEP, pre‐exposure prophylaxis.

The POWER implementation study was undertaken in Cape Town, South Africa, to develop scalable PrEP delivery strategies for AGYW (aged 16−25 years). From June 2017 to September 2020, 787 AGYW were enrolled, with study procedures described previously [24]. The Cape Town site was based in townships and set up a community‐based differentiated PrEP delivery model which included a mobile clinic, a government health facility, courier PrEP delivery and a youth PrEP club option (Figure 1).

Before the study commenced, ethical clearance was provided by the University of Washington and the University of Cape Town. Participants’ written informed consent was obtained in English or Xhosa prior to data collection, with parental consent waived for participants 16 and 17 years old.

### 2.2 PrEP delivery platforms

In this PrEP delivery model, participants could use the mobile clinic and government health facilities for PrEP initiation or refills, while the courier service and youth club provided PrEP refills only. Participants’ use of and movement between the four PrEP delivery platforms were tracked biometrically (via an electronic fingerprint reader) [25]. The community‐based mobile clinic provided PrEP (initiation and refills) as part of a nurse‐led integrated sexual and reproductive health (SRH) service, including hormonal contraception and point‐of‐care sexually transmitted infections (STI) testing, as previously described [25]. The government health facility provided public health services, including SRH and HIV services, and the POWER study team introduced and provided oral PrEP (for initiation and refill) to this facility, along with training on PrEP provision. A research administrator and two peer navigators from the study team were permanently placed at the facility to provide a friendly and fast‐tracked service to AGYW and ensure study data capturing. The youth PrEP club occurred on Saturdays at a community venue and was led by a nurse who provided additional education on PrEP and other SRH topics. It also created an engaging space for AGYW to share their PrEP use experiences with their peers. Participants accessing the community‐based youth club could get their PrEP refills at this engagement in exchange for a completed HIV rapid self‐test (HIVST) kit provided at the venue. The courier PrEP refill delivery option was fulfilled in collaboration with Iyeza Health^1^ (a community‐based courier service delivering chronic medication) and included two‐way communication via the Iyeza delivery App, which provided delivery reminders and allowed the participant to change the delivery date or location. Courier delivery included a pre‐packaged unmarked box including a PrEP refill, a home pregnancy test, a rapid HIVST kit with instructions and a referral mechanism should the test result be positive. The feasibility and acceptability of using the HIVST in this population had previously been confirmed [26].

In this PrEP delivery model, AGYW could use the mobile clinic and government health facilities for PrEP initiation or refills, while the courier service and youth PrEP club provided follow‐up and refills only.

### 2.3 Subset with in‐depth qualitative interviews

A subset of AGYW was purposively selected for in‐depth interviews (IDIs) at the end of the study period. Using convenience sampling, qualitative participants were recruited for IDIs if they had continuously used PrEP for at least 3 months, based on pharmacy records, and used more than one of the four PrEP delivery platforms for their PrEP refills.

### 2.4 Data collection

Qualitative interviews followed semi‐structured guides aimed at exploring the determinants of AGYW's choice and retention for each of the PrEP delivery platforms. Interview guides included questions related to their motivation to use a specific PrEP delivery platform, facilitators and barriers of each of the delivery platforms, and exploring the influence of sex partners, people the young women live with and peers in their decision to access PrEP from a particular platform (Supporting Information 1). The IDIs were conducted face‐to‐face in either English or isiXhosa, based on participant preference, by experienced social science interviewers who were independent of the study's clinical team. The IDIs lasted between 30 and 60 minutes and were audio recorded, then simultaneously translated and transcribed, and the qualitative team checked English transcripts for accuracy.

### 2.5 Data analysis

The codebook was iteratively created to reflect participants’ preferences, decision‐making and habits related to PrEP access from the various delivery platforms. Additional codes were included to capture themes, such as the role of disclosure, relationship dynamics, PrEP perceptions, social support and lifestyle in PrEP use decision‐making. A three‐person analysis team coded transcripts in Dedoose (Version 9.0.54, Los Angeles, CA: Socio‐Cultural Research Consultant, LLC). Sections of independently coded transcripts were periodically compared throughout the analysis process, with an average kappa of 0.80, indicating high reliability. Thematic analysis was applied to interpret AGYW's daily lives and PrEP access behaviours, which included a reflection on the barriers and facilitators AGYW experienced at each of the PrEP delivery platforms. Latent‐level themes [27] emerged from the data describing AGYW PrEP‐users preferences and decision‐making related to chosen PrEP delivery platforms and access.

## 3 RESULTS

### 3.1 Demographic and behavioural characteristics of sample

Participants in the POWER qualitative sub‐study in Cape Town included 26 AGYW aged 16−25 (median age = 20), who primarily lived with their parents (69.2%) or other family members (23%). All AGYW in this cohort had a current sex partner and were eligible for PrEP use. All young women in this cohort used hormonal contraception, with most (84.6%) using an injectable method needing regular (every 2 or 3 months) engagement with either the mobile clinic or government facility. Participants accessed PrEP from a combination of delivery platforms, with most using the mobile clinic (92.3%) and courier PrEP delivery (65.4%), followed by the government health facility (34.6%) and youth PrEP club (23.1%). HIVST was used during this study by 76.9% of participants to assess their HIV status at a PrEP refill.

### 3.2 PrEP service delivery platform decision‐making

Many (*n* = 16, 61.5%) participants mentioned the pervasive stigma and misinformation surrounding PrEP in their communities and how it affected their PrEP access and use. While the decision to use PrEP was highly personalized, AGYW who initiated and continued use had disclosure in common. Everyone in this cohort disclosed their PrEP use to a friend, while 88.5% disclosed to the people they lived with and 42.3% to a sex partner (Table 1). During the interviews, young women indicated that disclosure to the people they live with is important for adherence to the daily PrEP regimen. AGYW also shared their experiences of disclosure to sex partners, at times negotiating PrEP disclosure by framing it within the context of their HIV vulnerability due to high sexual violence in the community. Disclosure (whether to a sex partner or to people AGYW lived with) seemed to be important for continued PrEP use but, in most instances, did not influence AGYW's access to any of the PrEP delivery platforms. This was especially relevant in this cohort, who did not reside with their sex partners and experienced the burden of SRH prevention decisions on themselves.

**Table 1:** Demographic and behavioural characteristics of participants (*N* = 26)

	*N* (%)
**Age**	
16−19	11 (42.3)
20−25	15 (57.7)
**Living situation**	
Parents	18 (69.2)
Other family	6 (23.1)
Sex partner	0 (0.0)
Other	2 (7.7)
**Disclosed PrEP use to** ^a^	
People she lives with (parents/family)	23 (88.5)
Friend	26 (100.0)
Sex partner	11 (42.3)
**PrEP delivery platform (ever used)** ^b^	
Mobile clinic	24 (92.3)
Government facility	9 (34.6)
Courier delivery	17 (65.4)
PrEP club	6 (23.1)
**HIV self‐test (HIVST) experience**	
Yes (used HIVST to assess HIV status during the study)	20 (76.9%)
No	6 (23.1%)
**Hormonal contraception use**	
Injectable	22 (84.6)
Implant	2 (7.7)
Oral	2 (7.7)

Abbreviations: AGYW, adolescent girls and young women; PrEP, pre‐exposure prophylaxis.

^a^ Adolescent girls and young women (AGYW) could mark more than one category.

^b^ AGYW accessed multiple PrEP delivery platforms based on dispensing records.

*“Even now, he stays far, so it's easier for me to do my things.”* (Ayanda, 18‐year‐old).

*“…because guys do not even pay attention to whether you are on contraceptives or not, they only pay attention to what they want at that moment… So, I would say that the girls are the ones who [need to] look out for themselves, yes.”* (Grace, 18‐year‐old)

A recurrent theme in the interviews was that AGYW's selection of a PrEP delivery platform depended on their needs at a specific time, driving PrEP interest while weighing the best fit of the platform choice into their lives.

*“It depends on what you want for your life as a young woman, you will know where to get PrEP when you want to use it.”* (Avuyonke, 21‐year‐old)

**Figure 2:**
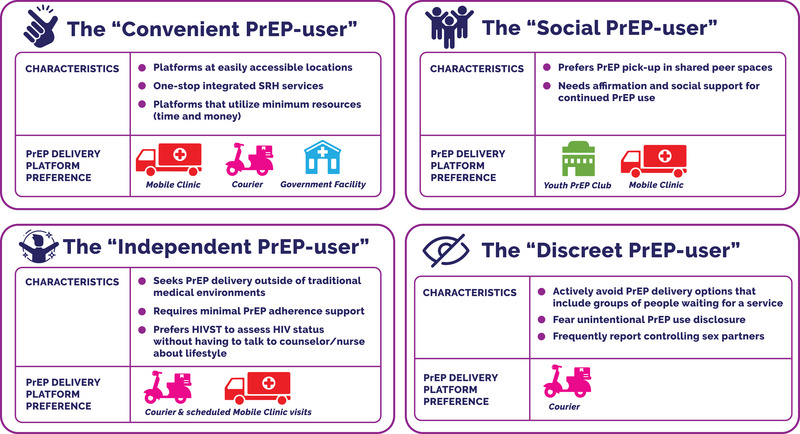
AGYW PrEP‐user segments and their PrEP delivery platform preferences. AGYW, adolescent girls and young women; PrEP, pre‐exposure prophylaxis.

Analysis of this AGYW cohort's perceived access barriers and facilitators to each of the four PrEP delivery platforms produced themes surrounding access convenience, privacy, the role of peers in continued PrEP use and AGYW's need (or lack thereof) for comprehensive, integrated services with elaborate PrEP use counselling compared to a simplified light touch quick approach to PrEP refills. Based on themes, we explored potential PrEP‐user segments that may highlight this AGYW cohort's needs, resources, relationships and values. The present here are four potential AGYW PrEP‐user segments: the convenient, the social, the independent and the discreet PrEP‐user (Figure 2).

Segmentation of AGYW PrEP‐users highlighted four profiles, including the convenient, the social, the independent and the discreet PrEP‐user.

### 3.3 The Convenient PrEP‐user

The “Convenient PrEP‐user” (the most common segment in this sample) preferred PrEP delivery at easily accessible locations, providing quick and/or integrated service with contraception and PrEP refills in a single visit. For the convenient PrEP‐user, using little time and financial resources to access PrEP was the highest priority. AGYW in this user segment most often used the mobile clinic available in their community at locations they frequent, such as schools, or courier delivery.

*“It's because it's much closer to my township. So, I'd usually walk when I'm going there. So, it was easy for me in that way. Cause I don't even need transport.”* (Thando, 17‐year‐old)

*“And the truck was like literally there outside my school, so I didn't have to travel for it, that was easier.”* (Ayanda, 18‐year‐old)

*“[Schools] that's where most young kids are, so they will get it easily.”* (Thando, 17‐year‐old).

The mobile clinic was also deemed convenient for access to integrated SRH services without long queues and expedited PrEP refill visits.

*“They give you your PrEP pills as well as contraceptives if you are using them. You don't wait for long without being attended to.”* (Lethu, 22‐year‐old)

*“When you got there you knew that you wouldn't have to wait – you get there, and they've already prepared for you so that you can quickly get them [PrEP refill] and leave.”* (Aviwe, 24‐year‐old)

The government health facility was also perceived as an acceptable platform for integrated contraception‐PrEP delivery if the facility was near where young women resided.

Courier PrEP delivery conveniently brought PrEP refills to a specified destination such as participants’ homes (or other suitable locations) and did not move around like the mobile clinic. Participants also shared instances of shifting to the courier option when they relocated further away from the mobile clinic routes to attend a different school or university and during the COVID‐19 lockdown periods.

*“When I had to come on my date, maybe I would not have money for transport firstly, then secondly you would find that we are busy with life, and I wouldn't be able to make time to come here. So, it's nice there because even if I am at a friend's place, or wherever I am…then at least they will transport them for me, and I can then take them… And also, during the Corona thing I also used the courier service.”* (Busisiwe, 19‐year‐old)

### 3.4 The Social PrEP‐user

The “Social PrEP‐user” sought PrEP delivery in shared peer spaces such as youth clubs or adolescent‐friendly mobile clinics, that provided affirmation and social support for continued PrEP use. This group normally disclosed their PrEP use, however, for the purpose of validation of their PrEP use, with some needing social approval even before making the decision to initiate PrEP. The social PrEP‐user valued opportunities to discuss adherence solutions and how PrEP was fitting into their lives and intimate relationships. Receiving PrEP in a peer environment at the youth clubs was seen as a space where they could overcome their concerns about community PrEP stigma.

*“there are a lot of things we'd discuss, you see? Things that you weren't able to admit to your friends, but when you hear someone say something you were afraid to say, you also get that relief, you see?”* (Sammy, 24‐year‐old)

*“It was fun and you can freely talk about anything. There was no one who can judge you or laugh at you. We were free to talk about anything. Healthcare workers [at mobile clinic] know how to work well with young people.”* (Lethu, 22‐year‐old)

Some AGYW also preferred going for their PrEP refills with a friend or female family member, such as a sister or cousin, and at times encouraged their friends to start taking PrEP leading to greater perceived PrEP adherence support.

*“Yes, my friends actually, they also actually went to take PrEP at some point. And then the support like that they also gave me is …I knew someone there knew that I was taking PrEP, so they would remind me (giggles) you see. And also like if there was something that I didn't understand sometimes, then they would also accompany me to the truck [mobile clinic] when I am fetching my things, then we can also ask the question together.”* (Lusanda, 16‐year‐old)

Lastly, they appreciated the comradery of everyone being at the youth club for the same reason, in contrast to government facilities.

*“Maybe, because there I will have time to meet other people who take PrEP, and we can discuss on our experiences and give each other advice.”* (Lethu, 22‐year old)

*“Cos, if we're at the club, we're all brought together by PrEP, and we know what we are there for. Where(as) people are at the clinic for different reasons, some are there to look at you, the club is better.”* (Thando, 17‐year‐old)

### 3.5 The Independent PrEP‐user

The “Independent PrEP‐user” preferred PrEP delivery that was outside of traditional medical environments, allowing for a high level of control over PrEP refill times. The independent PrEP‐user required minimal adherence support, believing that they can consistently and accurately assess their HIV vulnerability without the need to speak to a counsellor about sexual behaviour and relationship dynamics. Some AGYW in this group also liked using an HIV self‐test, indicating that they valued knowing their HIV status and appreciated that they could do it at home in their own time.

*“…the privacy, you do things alone and you are doing your thing. And no one is asking you certain questions that are unnecessary that you have to answer. Now you just do your thing, then take pictures and send it to them and then continue eating PrEP and then you go about your day.”* (Grace, 18‐year‐old)

*“Another thing that motivated me was the HIV self‐testing thing, I wanted to know my status and I could learn how to test myself.”* (Avuyonke, 21‐year‐old)

Furthermore, the independent PrEP‐user was often employed or busy looking for work, making it impossible to access government health facilities and mobile clinics with limited hours of operation.

*“If you're a person who's working, right? Let's say you start work at 8 in the morning and you leave at 5, you won't be able to meet up with them [clinic]. What made the delivery easy was that they're able to even deliver at your workplace.”* (Ncumisa, 24‐year‐old).

*“What was difficult was when the clinic would be full, and there would be many of us… when they deliver it's a quick process for myself as well as them.”* (Grace, 18‐year‐old)

Young women in this category primarily chose the PrEP courier delivery option. They appreciated the advance contact with the courier service, allowing them to change or reschedule deliveries to a convenient time and/or location that will fit into their schedule and cut down on travel time and costs.

*“The people [courier delivery] tell you when they are coming to you and you can say I'm not available you can come at a different time. They go according to your schedule.”* (Avuyonke, 21‐year‐old)

### 3.6 The Discreet PrEP‐user

The “Discreet PrEP‐user” was most concerned about stigma and people's perception of her as a PrEP‐user, including her peers. Young women in this segment hardly disclosed their PrEP use and definitely not to their sex partners (often described as very controlling) which also led to skipping PrEP doses when with their partner. How people perceived them engaging with a specific delivery platform played a role in how and when they accessed PrEP. The discreet PrEP‐user avoided delivery options that had groups of people waiting for services such as at the government health facility, or where unintentional PrEP use disclosure was evident like at the PrEP youth clubs.

*“Sometimes there are children that come for their family planning, you will be shy to take PrEP in front of them. They will say you are taking PrEP in a nasty way even though you are doing the right thing.”* (Ncumisa, 24‐year‐old)

Discreet PrEP‐users accessed integrated SRH services where they could conceal their PrEP refill visit within their contraception appointment, but they mostly appreciated the privacy of the courier delivery.

*“I was spotted going to collect pills in the clinic not knowing what pills I went to collect. But, when they spot the [courier delivery] car, they'll just ask what the car was here for, but they can't see what's inside [the box], and what we have [PrEP].”* (Yolanda, 20‐year‐old)

However, due to their heightened sensitivity to stigma and avoidance of disclosure, discreet PrEP‐users will avoid and reschedule a courier PrEP delivery when there is any risk of unintentional disclosure.

*“If the delivery person would call, and I am hanging out with people, I would silence my phone, but at the back of my head, I'll be thinking about how I need PrEP as I have run out… I'd answer if I'm on my own.”* (Didi, 18‐year‐old)

## 4 DISCUSSION

This qualitative exploration of AGYW continuous PrEP‐users in South Africa highlighted four potential behavioural profiles (independent, convenient, social and discreet PrEP‐users) related to their engagement with specific PrEP delivery platforms. Although all the young women in this cohort reported an inclination towards continued PrEP use, their level of autonomy, resource constraints experienced, and the influence of significant others, shaped their access to PrEP services. Mobile clinics were the most used PrEP delivery platform, accessed by 92% of AGYW in this cohort. Mobile clinics have been described as acceptable and convenient for AGYW, especially those from under‐resourced areas, by providing PrEP services at convenient times, at easily accessible locations and integrated with contraception provision for optimal access [25, 28, 29]. The second most popular PrEP delivery platform was courier delivery, which AGYW selected for its privacy, convenience and limited interaction needed, especially by AGYW considered to have a demanding lifestyle schedule or those avoiding PrEP use disclosure. The youth PrEP club, similar to other demonstration studies, showed that supportive peer environments may increase adherence to PrEP, while also overcoming community stigma [30, 31, 32]. However, the PrEP club had a lower level of use compared to the other platforms and may be suited for a targeted sub‐sample of AGYW. While the presence of peer navigators promoted AGYW accessing integrated SRH and PrEP services at the government health facility, this platform was still criticized for its lack of privacy and long waiting times, similar to other studies [33, 34, 35]. Comfort with HIVST had minimal influence on PrEP delivery choice and was highly acceptable [36, 37], especially by the independent and convenient PrEP‐users.

Previous research suggests that PrEP use in AGYW is influenced by both internal drivers and external circumstances [11]. Beyond that, we found that these intrinsic and extrinsic motivations along with AGYW's lifestyle habits also translate to their choice of PrEP delivery platform. AGYW in this study found themselves on a continuum of intrinsic (strong self‐determination and autonomy finding PrEP adherence personally fulfilling) and extrinsic motivation (where PrEP use habits are influenced by external factors of either rewards, social pressure or fear of negative consequences) [38, 39, 40]. Intrinsic motivation was most notable in the independent PrEP‐user seeking PrEP delivery that fit into their schedule and require minimal adherence support. Previous research primarily associates independent PrEP use with men who have sex with men and transgender populations [41, 42, 43, 44, 45, 46, 47]; however, our findings suggest that some AGYW might also be suited for simplified, automated PrEP services, which in this study was courier delivery but could include PrEP delivery models that use telemedicine and pharmacies [14]. Extrinsic motivation was a factor for social PrEP‐users, seeking peer‐supported PrEP services that provide affirmation for continued PrEP. While community PrEP stigma was a crosscutting theme in the study cohort, the discreet PrEP‐user especially avoided delivery options with a risk of unintentional disclosure, fearing stigma, discrimination or adverse consequences from controlling sex partners [48, 49]. While the discreet PrEP‐user preferred courier delivery, similar to AGYW with low disclosure who struggles with consistent PrEP adherence [7, 50, 51, 52], this group at times missed PrEP refill visits when it threatened unintentional PrEP use disclosure. While it is generally accepted that people who display intrinsic motivation for their health behaviour display better persistence [38], our findings suggest that the PrEP delivery mechanism can be designed to also promote continued PrEP use in extrinsically motivated PrEP‐users—in this instance by providing high social reward services (youth PrEP club) or extremely private services (courier PrEP delivery and HIVST for discreet PrEP‐users).

AGYW are diverse in their lifestyles, needs, habits and levels of influence from those around them, which in this study influenced their engagement with service delivery platforms for both uptake and effective continued use of PrEP [53]. Understanding different market segmentations and how these influence AGYW's PrEP access will allow for tailored demand creation that creates the greatest likelihood that people will be connected to PrEP delivery platforms acceptable and feasible to them [18, 21]. In addition, when this information is made available to healthcare providers, adherence counselling can be geared towards AGYW's preferences and trade‐offs regarding delivery platforms for PrEP refill visits for optimum use by all AGYW. This will also be important during the introduction of new biomedical PrEP modalities within a choice framework of HIV prevention options, where some methods (such as long‐acting injectable cabotegravir) will be confined to a few delivery platforms where a healthcare provider can administer the therapy, while other long‐acting methods such as the dapivirine vaginal ring will be amenable to multiple delivery platforms outside traditional facilities, including courier delivery, reinforcing convenience, independence and discretion [54]. In the rollout and scaling up of HIV prevention for AGYW, implementers are encouraged to consider both PrEP modality and PrEP delivery platform preferences. Successful market segmentation is a multi‐phase process. The next step would be to further differentiate and validate these PrEP‐user segments through a quantitative investigation of AGYW demographic and psychographic characteristics associated with these profiles. In addition, further research into cost‐effectiveness and evaluation of PrEP service delivery options that can engage large populations of AGYW may be beneficial.

### 4.1 Limitations

The primary limitation of this study is its qualitative design with a small sample of 26 AGYW, and therefore, caution needs to be applied in generalizing the results beyond this specific study population. In addition, the sample consisted of AGYW who had 3 months of sustained PrEP use, and different segmentation might apply to AGYW who struggle to use PrEP consistently or those who were interested in PrEP but did not initiate from any of these PrEP delivery platforms. Also, this sub‐study had a relatively short follow‐up period (∼ 3 months), and different segmentation might apply to long‐term continued PrEP‐users. Furthermore, the study cohort primarily lived with their parents or other family and, therefore, experienced low influence from sex partners on their PrEP delivery platform choice—this might look different in populations residing with their sexual partners.

## 5 CONCLUSIONS

This qualitative investigation of factors influencing AGYW's preferred PrEP delivery platform illuminated four potential key behavioural profiles: the independent, convenient, social and discreet PrEP‐user. By segmenting the market, we gain a deeper understanding of the different types of AGYW PrEP‐users, enabling the development of targeted messaging and campaigns, as well as inclusive service delivery mechanisms, to best reach specific segments and result in sustained PrEP use and HIV prevention benefits for all AGYW who may benefit from PrEP.

## COMPETING INTERESTS

ER, JMB, CC and L‐GB report personal fees from Gilead Science outside the submitted work. JMB is employed by Gilead Sciences, Inc.

## AUTHORS’ CONTRIBUTIONS

Funding acquisition: CC, JMB and RFJ. Study design and investigation: CC, JMB, ER and L‐GB. Project administration: RFJ, JFM and ER. Data analysis: ER, KM and RFJ. Writing—original draft: ER. Writing—review and editing: KM, RFJ, KJS, JMB, CC, L‐GB, RH, RJ, JFM and GO.

## FUNDING

The research leading to these findings received funding from USAID (AID‐OAA‐A15‐00034). PrEP (Truvada) was sponsored by Gilead Sciences Inc.

## Supporting information

File S1: In‐depth interview (IDI) guide for young women. Pdf with interview guide used for data collection during in‐depth interviews.

## Data Availability

The data that support the findings of this study are available on request from the corresponding author. The data are not publicly available due to privacy or ethical restrictions.

## Footnotes

1
www.iyezahealth.co.za
