# Supplementary material for: Exploring adolescent girls and young women's PrEP‐user profiles: qualitative insights into differentiated PrEP delivery platform selection and engagement in Cape Town, South Africa
Source: J Int AIDS Soc. 2024 May 2;27(5):e26254. doi: 10.1002/jia2.26254 (PMC11063778; doi:10.1002/jia2.26254)
Supplement: Supplementary file 1 — File S1: In‐depth interview (IDI) guide for young women. Pdf with interview guide used for data collection during in‐depth interviews. [file JIA2-27-e26254-s001.pdf]

Supporting Information file 1: In-depth Interview (IDI) guide for young women

Opening Remarks

*[**Purpose:** The intent of this portion of the guide is to welcome the participant and make them as comfortable as possible by explaining the IDI and letting them know what to expect from the experience. The interviewer can also remind the participant about confidentiality procedures and explain how data will be dealt with (stored, transcribed and analyzed). Don't refer to this as an "in-depth interview". Instead call this a "chat" or "discussion."]*

Interviewer Reads: We are happy that you are here to give us your experiences and advice on how best to deliver PrEP to young women in your community. As we mentioned in the informed consent, our goal is to have discussions with people who have experience with PrEP and the POWER study. We will ask about your experiences of the POWER study and using PrEP. The opinions and experience you share during this discussion will give us information about what you would like in an HIV prevention product and service delivery. As we said, you are one of 30 people aged 16-25 who will be interviewed during this part of the POWER Study.

1. I'd like to start by first hearing a bit more about where you live. What is life like for a young woman in your community?
    - What are the highlights and what are the challenges?
    - Who influences sexual and reproductive health decisions for young women (such as whether to have sex; contraception use; HIV prevention)

2. What have you heard people say about PrEP in your community (if anything)?
    - Is there widespread knowledge/ awareness of PrEP/ PrEP research or other medical research in the community?
    - What are your reactions to what you have heard? How does this affect you?
    - What made you decide that PrEP was right for you?

3. In this study you are allowed to pick up your PrEP refills from different platforms including the Tutu Teen Truck, the Weltevreden clinic/local DoH clinic, the POWER adherence club, and the Iyeza courier service. Which of these PrEP pick-up platforms did you use and what motivated you to use this specific method?
    - How easy/difficult was it to access these services?
    - What are some of the challenges/barriers that you experienced which made it difficult for you to access this platform?
    - How did you overcome these challenges? What strategies did you try to ensure that you take your PrEP / come to study visits?
    - What are some of the things that made it easier/helped you to access/take the PrEP?
    - Overall, what would you say are the pros and cons of the specific platform you used?

4. Did you tell anyone that you were taking PrEP?
    - Is there a reason you told someone / did not tell anyone about it?
    - Did you receive any support from the person you told? What kind of support did you receive? How did that affect your PrEP use?
    - Did you receive any negativity from person that you told, what did they say? How did that affect your PrEP use?
    - How did the level of support you received affect which of the PrEP pick up platforms you used?

5. Tell me a bit about your living situation. Please describe the people you live with in your household.
    - Who do you live with?
    - What's your relationship to them?
    - What aspects of your home made it easy or difficult to use PrEP?
        - How did the amount of privacy or lack of privacy you had affect your use?
        - How did the people who you live with influence your use?
    - How does your living situation/people you live with influence which PrEP pick up platform you access?

6. I would also like to hear about your relationships. Let's start by having you think about your current relationship. What is it like?
    - How and where did you meet?
    - What attracted you to him/her?
    - What does your family think about your relationship?
    - What do your friends think about the relationship?
    - In your relationship who makes decisions about having sex? (incl. type of sex, timing of sex, frequency of sex, condom use)
    - Can you describe a situation where you made a decision around sex with your partner?
    - [*If they've had more than one partner*] How does it differ with different types of partners (e.g. casual, one night stand, long term, paying client)?
    - How does your partner influence your use of PrEP? Does your partner know you are taking PrEP? If so, what is your partner's opinion of PrEP?
    - How does your relationship influence which PrEP pick up platform you access?

7. Can you imagine yourself ever using one of the other PrEP pick-up platforms (name the ones the participant did not use)?
    - If yes, can you describe the scenario in which you could imagine wanting to use one of these services?
    - If no, what are the barriers/challenges in using each of the other PrEP pick-up platforms?

8. Where else do you think PrEP should be made available to young women like yourself?
    - Where would be most convenient or comfortable for you to get it?

- What do you suggest other young women should do to successfully access and take PrEP?
